# Cost-benefit analysis of haemodialysis in patients with end-stage kidney disease in Abuja, Nigeria

**DOI:** 10.1186/s13561-024-00529-z

**Published:** 2024-07-03

**Authors:** Yakubu Adole Agada-Amade, Daniel Chukwuemeka Ogbuabor, Eric Obikeze, Ejemai Eboreime, Obinna Emmanuel Onwujekwe

**Affiliations:** 1 Department of Health Administration and Management, University of Nigeria, Enugu Campus, Enugu State, Nigeria Enugu, Enugu, Nigeria; 2 National Health Insurance Authority, Abuja, Nigeria; 3 Department of Health Systems and Policy, Sustainable Impact Resource Agency, Enugu, Nigeria; 4 Health Policy Research Group, Department of Pharmacology and Therapeutics, College of Medicine, University of Nigeria Enugu Campus, Enugu, Nigeria; 5 Department of Psychiatry, Faculty of Medicine, Dalhousie University, Halifax, NS Canada

**Keywords:** Cost-benefit analysis, Contingent valuation method, Benefit-cost ratio, Economic evaluation, Nigeria

## Abstract

**Background:**

Significant gaps in scholarship on the cost-benefit analysis of haemodialysis exist in low-middle-income countries, including Nigeria. The study, therefore, assessed the cost-benefit of haemodialysis compared with comprehensive conservative care (CCC) to determine if haemodialysis is socially worthwhile and justifies public funding in Nigeria.

**Methods:**

The study setting is Abuja, Nigeria. The study used a mixed-method design involving primary data collection and analysis of secondary data from previous studies. We adopted an ingredient-based costing approach. The mean costs and benefits of haemodialysis were derived from previous studies. The mean costs and benefits of CCC were obtained from a primary cross-sectional survey. We estimated the benefit-cost ratios (BCR) and net benefits to determine the social value of the two interventions.

**Results:**

The net benefit of haemodialysis (2,251.30) was positive, while that of CCC was negative (-1,197.19). The benefit-cost ratio of haemodialysis was 1.09, while that of CCC was 0.66. The probabilistic and one-way sensitivity analyses results demonstrate that haemodialysis was more cost-beneficial than CCC, and the BCRs of haemodialysis remained above one in most scenarios, unlike CCC’s BCR.

**Conclusion:**

The benefit of haemodialysis outweighs its cost, making it cost-beneficial to society and justifying public funding. However, the National Health Insurance Authority requires additional studies, such as budget impact analysis, to establish the affordability of full coverage of haemodialysis.

**Supplementary Information:**

The online version contains supplementary material available at 10.1186/s13561-024-00529-z.

## Background

Nigeria established the National Health Insurance Scheme in 1999. At inception, the benefit package excluded renal replacement therapy, such as kidney transplant and haemodialysis, for managing end-stage kidney disease (ESKD). ESKD is the last chronic kidney disease stage, losing 85–90% of function [1]. Haemodialysis is a medical procedure in which blood circulates through an external machine with a solution of prescribed electrolyte composition and semipermeable membrane, filtering waste products and excess fluid from the blood of end-stage kidney disease patients [2]. In 2012, the National Health Insurance Authority (NHIA), then called the National Health insurance Scheme (NHIS), integrated partial coverage of haemodialysis into the Formal Sector Social Health Insurance Programme’s benefits [3, 4], but the implementation commenced in 2014. The partial coverage meant that the health insurance agency bore the cost of six haemodialysis sessions annually, while ESKD patients paid for any other sessions.

The burden of ESKD and the demand for haemodialysis are increasing. In Sub-Saharan countries of Africa, the prevalence of chronic renal diseases is between 10.7% and 13.9% [5]. In Nigeria, 20.4% of 14,253 screened participants had chronic kidney disease [6]. The estimated annual percentage change in the summary exposure value of kidney dysfunction ranges from 0.31 to 0.51 in sub-Saharan Africa [7]. Likewise, healthcare costs are growing. The estimated annual cost per patient for dialysis ranges from about $3,424 to $47,971 in LMICs [8]. Direct medical costs, especially those for medications and consumables, are the main drivers of the haemodialysis cost. Therefore, the cost of managing ESKD imposes a significant financial burden on patients, households, and society.

A session of haemodialysis costs about $150 ($62 and $250), which exceeds Nigeria’s 2018 minimum wage of N18,000 ($57.2) [9, 10]. Hence, paying out-of-pocket for haemodialysis at the point of service increases the likelihood that ESKD patients will experience financial hardship or impoverishing health expenditure. Most ESKD patients in Nigeria can only afford dialysis up to two to three months of initiation [11]. Given the economic burden of haemodialysis on households, stakeholders are advocating for the full coverage of haemodialysis by the NHIA. However, economic evidence informing the inclusion of haemodialysis in the benefits package of the social health insurance scheme is lacking. Moreover, the cost-benefit analysis did not inform NHIA’s initial decision to cover haemodialysis partially. Therefore, the social value of the partial coverage of haemodialysis was uncertain.

Significant disparities exist in the financing of chronic kidney disease. Most high-income and upper-middle-income countries fund chronic haemodialysis through the government, whereas few LMICs publicly fund chronic haemodialysis [12]. Government-funded dialysis is associated with higher access to dialysis than self-funded out-of-pocket (OOP) [13]. In low-resource settings, managing ESKD relies heavily on out-of-pocket payment (OOP), significantly hindering access to haemodialysis [10, 14]. Long-term dialysis is only sustainable with public subsidy since ESKD patients cannot afford to pay for dialysis beyond a few months [11, 12].

Nevertheless, many LMICs are considering expanding their social health insurance benefits to cover haemodialysis and ensure financial protection for ESKD patients [13]. In countries such as Rwanda, Thailand, China, Colombia, and India, social health insurance schemes have improved the affordability of dialysis among ESKD patients [15–19]. However, insurance ownership might not eliminate all costs. Despite China’s health insurance coverage of dialysis, out-of-pocket spending among ESKD patients in rural areas is up to 45% of the cost of care, increasing their likelihood of financial hardship [20]. While integrating haemodialysis fully into the social health insurance benefits using public subsidies might reduce OOPS and protect ESKD patients from financial hardship, evidence that full coverage of haemodialysis is socially worthwhile and justifies public funding is scarce in most sub-Saharan African countries.

Cost-benefit analysis (CBA) of haemodialysis implies an implicit social value of maintaining ESKD patients on haemodialysis, that is, an estimate of the price society would pay to preserve the lives of ESKD patients [21, 22]. An intervention with an increased social value, that is, its benefits outweigh the costs, is preferable to an intervention with low social value [22]. Cost-benefit analysis is critically suited for valuing goods and services with health and non-health consequences [1]. Sustainable access to haemodialysis improves quality of life and longevity of patients with ESKD [23]. Hence, CBA would be applicable in valuing the consequences of haemodialysis using monetary units.

The study assessed the net monetary benefit of haemodialysis compared with comprehensive conservative care (CCC). Prior to integrating partial coverage of haemodialysis, NHIA’s benefits package included only comprehensive conservative care for managing ESKD patients. Comprehensive conservative care mainly consists of managing symptoms and using interventions (aside from dialysis) to slow the progression of kidney disease and reduce the risk of complications or adverse events. [13]. Our study is the first study analyzing the cost-benefit of haemodialysis among ESKD patients in Nigeria. This study, therefore, provides decision-makers evidence of the costs and benefits that enable them to decide whether publicly funded haemodialysis through the social health insurance scheme is worthwhile.

## Methods and materials

**Fig. 1:**
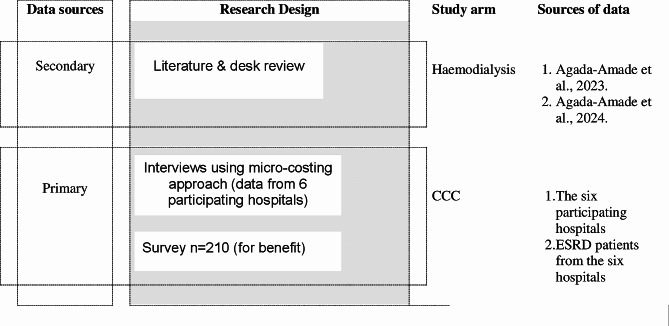
Schematic of the study methods and data sources

The data sources, research design, study arm and sources of data in this study are summarized in Fig. 1. The study adopted an ingredient-based costing approach (a mix of primary and secondary analysis). Additional file S1 includes the costing template.

### Costs of haemodialysis

We abstracted the haemodialysis costs from a previous study in Abuja, Nigeria [4]. In the primary study, the costing was conducted from a provider and patient perspective using a micro-costing approach [24]. The provider costs were both fixed and variable components. The fixed costs comprised building and equipment. The study assessed the value of the dialysis area by comparing its surface area to the entire hospital building. The fixed costs were discounted at a 3% rate. The variable costs consisted of staff salaries, drugs and consumables, and overheads such as electricity and water. The time staff spent on haemodialysis informed the estimates of staff salaries, while drugs and consumables were based on NHIA’s price list. The patient costs encompassed the direct out-of-pocket payments for transportation, feeding, drugs, and laboratory services procured monthly by the patients. We annualized the monthly patient costs by multiplying them by 12. The costs were estimated at the 2018 Nigerian Naira (N) to US dollars exchange rate: N308.5 to $1 and summarized using means and standard deviation.

### Benefits of haemodialysis

We abstracted the benefits of haemodialysis from a previous study in Abuja, Nigeria [25]. The study adopted the WTP approach, which determines the highest amount of money an individual is willing to pay from their income to enhance their health or lower their risk of dying [21]. Using a bidding strategy, the study asked the ESKD patients to state their maximum WTP for a haemodialysis if they must pay immediately to enhance their health or lower the risk of disabilities or death. The bidding game begins with a single bid and increases or decreases until the patient states the maximum willingness to pay (WTP). Initially, we asked the patients if they were willing to pay a specific amount, a higher cost, or a lower amount. Then we inquired about the maximum amount the patient would pay for haemodialysis. The monetary benefits were estimated at the 2018 Nigerian Naira (N) to US dollars exchange rate: N308.5 to $1. The monetary benefits were summarized using means and standard deviation.

### Cost and benefits of comprehensive conservative care

We undertook a cross-sectional survey in Abuja, Nigeria’s Federal Capital Territory (FCT), to collect data on the costs and benefits of comprehensive conservative care. Abuja is in the North-Central region of Nigeria and comprises six Area Councils and many satellite towns. The population was 2.9 million in 2018, with almost an equal gender composition. The two most common occupations of residents of Abuja are farming and public service. Abuja has the highest social health insurance enrolment in Nigeria. Also, Abuja has 15 dialysis centres serving the Federal Capital Territory and the entire North-Central region [10]. The survey included three (3) public, one (1) public-private partnership, and two (2) private dialysis centres chosen to optimize dialysis coverage, ownership variation, and geographic distribution of dialysis centres.

We surveyed CKD patients receiving CCC (*n* = 210). Given a 95% confidence limit, an acceptable error of 0.05, a 10% non-response rate, and a prevalence of chronic renal illnesses of 10.76, we calculated the minimum sample size for the survey (*N* = 165) using the sample size determination formula for infinite proportion [4, 25]. The study adopted a two-stage stratified sampling technique. We gathered data from July 2019 to February 2020. The costing was conducted from a provider and patient perspective and limited to direct medical and non-medical costs. The study utilized a micro-costing approach which employs detailed resource utilization and unit cost data to estimate precise economic costs [24]. The provider costs were both fixed and variable components. The fixed costs comprised building and equipment including sterilizer and laboratory equipment. The study assessed the value of the building area by comparing the surface area of the treatment unit to the entire hospital building. The fixed costs were discounted at a 3% rate. The variable costs consisted of staff salaries, overheads such as electricity and water, and drugs and consumables such as disposable masks, safety boxes, plastic aprons, waste disposal, laundry and laboratory commodities. The staff salaries were obtained by multiplying the gross salaries by the proportion of time staff spent annually providing CCC. The overheads were based on estimated annual consumption. The unit costs of drugs and consumables were derived from the NHIA’s price list or market when an item was not covered by NHIA. We estimated annual provider costs of drugs and consumables by assuming weekly visits per patient for CCC and multiplying the unit cost by 52 weeks. The patient costs encompassed the direct out-of-pocket payments for transportation, feeding, drugs, and laboratory services procured monthly by the patient. We annualized the monthly patient costs by multiplying them by 12. The costs were summarized using means and standard deviation. The costs of CCC were estimated at the 2018 Nigerian Naira (N) to US dollars exchange rate: N308.5 to $1.

We obtained the benefit using the maximum willingness to pay (WTP) method, a type of contingent valuation method (CVM), which determines the highest amount of money an individual is willing to pay from their income to enhance their health or lower their risk of dying [21]. The survey employed the bidding technique to ask the respondents to state their maximum WTP for comprehensive conservative care session if they must pay immediately. Before expressing their WTP for preventing a deterioration in health—that is, going from a better health condition to a worse one with all the limitations and, eventually, death—the respondents evaluated two different health states in each scenario [25]. Initially, we asked the patients if they were willing to pay a specific amount, a higher cost, or a lower amount. Then we inquired about the maximum amount the patient would pay for CCC. The monetary benefits were summarized using means and standard deviation. The monetary benefits of CCC were estimated at the 2018 Nigerian Naira (N) to US dollars exchange rate: N308.5 to $1.

Approval for conducting the survey was obtained from the Federal Capital Territory (FCT) Health Research Ethics Committee (FHREC/2019/01/02/10-01-19) and hospitals. Prior to data collection, the respondents gave their informed written consent. We anonymised respondents’ data and stored them safely in password-protected computer.

### Benefit-cost analysis

We estimated two benefit-cost outcome measures. The first outcome was the benefit-cost ratio of haemodialysis and CCC. The benefit-cost ratio (BCR) is the monetary benefit divided by the cost of a treatment option [21]. If the BCR exceeds 1, the treatment option is considered value for money. The equation for BCR is:

$$BCR=\frac{{B}_{X}}{{C}_{X}}$$

where B_X_ is the benefit of haemodialysis or CCC, and C_X_ is the cost of haemodialysis or CCC.

The second outcome measure was net benefit, which is the difference between the benefit and cost of a treatment option [21]. A positive benefit implies that the treatment option was cost-beneficial, and a negative benefit means the treatment option was not cost-beneficial to society. The Net benefit, $NB={B}_{X}-{C}_{X}$, where B_X_ is the benefit of haemodialysis or CCC and C_X_ is the cost of haemodialysis or CCC.

Our analysis used a Monte Carlo simulation to conduct a probabilistic sensitivity analysis (PSA), which helped us to consider joint uncertainties associated with different model parameters. Our probabilistic model included input parameters such as the costs related to Haemodialysis (HD) and Comprehensive Conservative Care (CCC). We conducted the simulation with the incremental annual net cost of haemodialysis over CCC as the base case. The PSA generated 1,000 random iterations for each input parameter and calculated the net benefits for both HD and CCC treatments. For the one-way and two-way sensitivity analysis, we varied the total costs of haemodialysis, fixed costs, provider costs, patient costs, WTP, and CCC within the 95% confidence intervals. Varying the costs allowed us to observe the impact of the minimum and maximum costs on the net benefits and BCRs.

## Results

### Cost estimates

According to Table 1, the unit costs for comprehensive conservative care and haemodialysis were USD 72.44 and USD 152.21, respectively (Table 2). The annual mean costs for comprehensive conservative care and haemodialysis were USD 3,540.87 and USD 23,744.76, respectively.

**Table 1:** Cost of haemodialysis and comprehensive conservative care (CCC)

Cost perspectives	Cost elements	Haemodialysis (*n* = 230)	CCC (*n* = 210)
		Cost in USD ($)	Cost in USD ($)
Provider	Variable cost	103.20	8.33
	Fixed	20.50	30.11
	Total provider cost	123.70	38.85
Patient	Direct cost	28.51	33.59
Unit cost per session		152.21	72.44
Annual cost per patient		23,744.76	3,540.87
Annual provider cost per patient		19,297.20	1898.99

### Benefit estimates

**Fig. 2:**
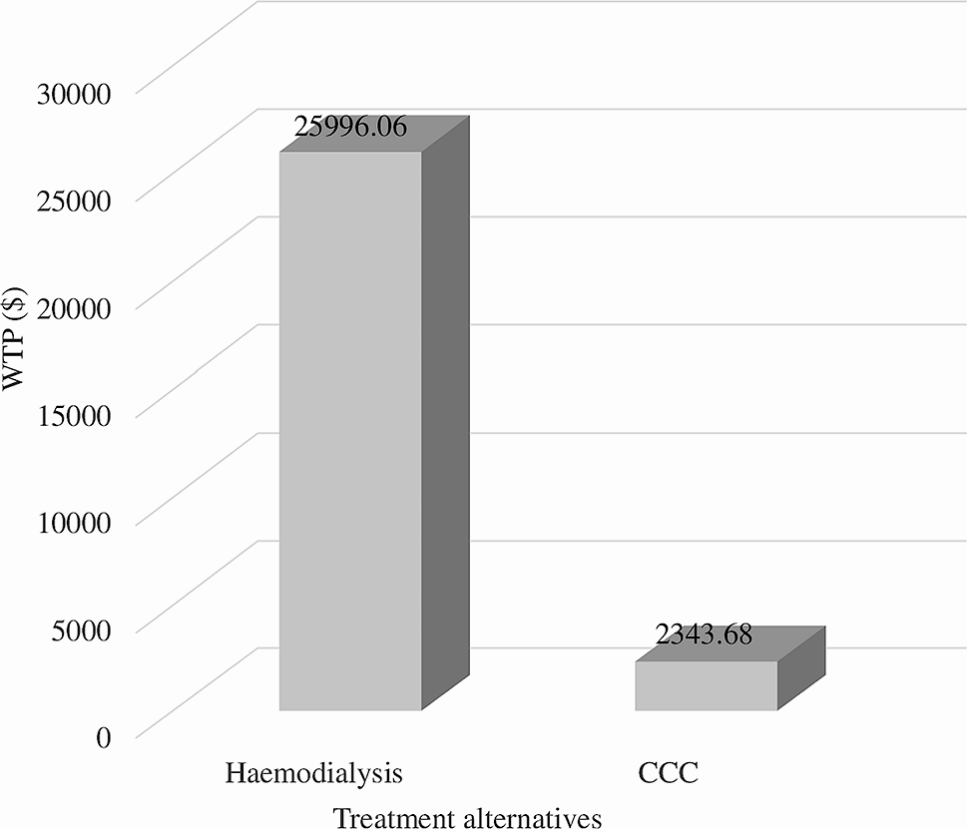
Benefits of haemodialysis and comprehensive conservative care

Patients were willing to pay a mean of USD 25,999.06 for haemodialysis, as Fig. 2 shows, whereas CCC patients were willing to pay a mean of USD 2,343.68 [23].

#### Benefit-cost ratios and net benefits of haemodialysis

Table 2 shows that the net benefit of haemodialysis (2,251.30) was positive, while the net benefit of CCC was negative (-1,197.19). The benefit-cost ratio of haemodialysis was greater than one, while that of comprehensive conservative care was less than one, implying that haemodialysis is more cost-beneficial than CCC. The benefit-cost ratio of haemodialysis (1.35) was also higher than that of comprehensive conservative care (1.23) when we considered only the provider costs, indicating a higher net benefit.

**Table 2:** Benefits of haemodialysis and comprehensive conservative care (CCC)

Cost perspective	CBA indices	Haemodialysis	CCC
Provider and patient	Benefit cost ratio	1.09	0.66
	Net benefit (USD)	2,251.30	-1,197.19
	BCR (%)	109	66
Provider cost alone	Benefit cost ratio	1.35	1.23
	Net benefit (USD)	6,698.86	444.69
	BCR (%)	135	123

### Sensitivity analysis

**Fig. 3:**
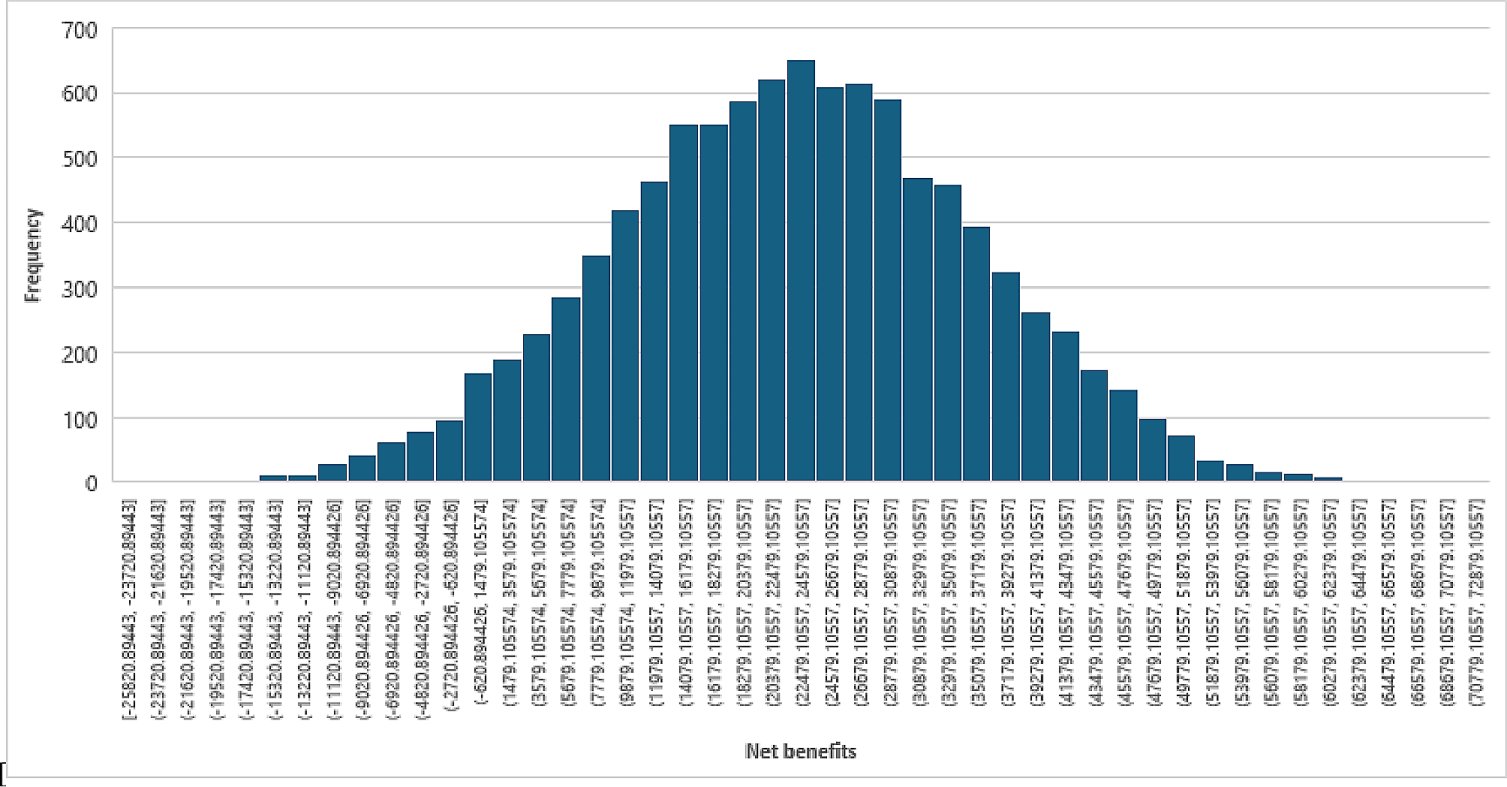
Probabilistic sensitivity analysis of cost-benefit of haemodialysis and CCC

As shown in Fig. 3, the probabilistic sensitivity analysis indicates that there is a 100% probability that haemodialysis will have incremental net cost benefit over comprehensive conservative care. Table 3 shows that the net benefit of haemodialysis remained positive, while the net benefit of CCC is negative. Furthermore, BCRs of haemodialysis mainly exceeded 1, implying that haemodialysis will be a profitable intervention for society. Conversely, the BCRs of CCC were mostly less than one, suggesting that CCC was less cost-beneficial than haemodialysis.

**Table 3:** Sensitivity analysis of the benefit-cost ratios of haemodialysis and CCC

S/*N*	Scenarios	Haemodialysis				CCC			
		Cost ($)	WTP ($)	BCR	Net benefit	Cost ($)	WTP ($)	BCR	Net Benefit
1	Base	23,744.76	25,996.06	1.09	2,221.30	3,540.87	2343.68	0.66	-1,197.19
2	Maximum total cost	25,500.59	25,996.06	1.02	495.47	3,831.10	2,343.68	0.55	-1,487.42
3	Minimum total cost	21,994.40	25,996.06	1.18	4,001.66	3,250.64	2,151.58	0.83	-1,099.06
4	Maximum fixed cost	24,704.16	25,996.06	1.05	1,291.90	3,660.09	2,343.68	0.64	-1,316.41
5	Minimum fixed cost	22,785.36	25,996.06	1.14	3,210.70	3,397.80	2,343.68	0.69	-1,054.12
6	Only provider cost	19,297.20	25,996.06	1.35	6,698.86	2,877.64	2,343.68	0.81	-533.96
7	Only patient cost	4,447.56	25,996.06	5.85	21,548.50	663.23	2,343.68	3.53	1,680.45
8	Maximum WTP	23,744.76	27,918.37	1.18	4,173.61	3,540.87	2,535.78	0.83	-1,005.09
9	Maximum total cost, maximum WTP	25,500.59	27,918.37	1.09	2,417.78	3,831.10	2,535.78	0.66	-1,295.32
10	Minimum total cost, minimum WTP	21,994.40	24,079.74	1.09	2,085.34	3,250.64	2,151.58	0.66	-1,099.06
11	Maximum total cost, minimum WTP	32,495.08	28,493.71	0.88	-4,001.37	3,831.10	2,151.58	0.56	-1,679.52
12	Minimum total cost, maximum WTP	21,994.40	27,918.37	1.27	5,923.97	3,250.64	2,343.68	0.72	-906.96
13	Altruistic WTP added to WTP	23,744.76	27,535.95	1.16	3,791.19	3,540.87	2,353.62	0.66	-1,187.25

## Discussion

The study compared the cost-benefit of haemodialysis versus comprehensive conservative care for ESKD patients using the cost of illness and stated willingness to pay approaches. The study revealed haemodialysis has a positive net benefit and benefit-cost ratio greater than one, implying that the benefits of saving lives and reducing the suffering of ESKD patients receiving haemodialysis exceeded that of comprehensive conservative care. Even when the study estimated the benefit-cost ratios with provider cost alone, haemodialysis had more social value than comprehensive conservative care. Therefore, publicly financing haemodialysis is cost-beneficial and socially worthwhile since an intervention with an increased social value is preferable to others [22]. In support of the findings of the current study, a previous Iranian study found that renal replacement therapy is cost-beneficial [1]. The government must consider policy options, including expanding insurance coverage with government subsidies for ESKD patients experiencing scarcity, providing dialysis through lower-cost public facilities, and harnessing altruistic donations.

The probabilistic sensitivity analysis is consistent with the findings from our base case analysis that haemodialysis is a more cost-beneficial option than CCC. The deterministic sensitivity analysis results demonstrate that BCR was sensitive to variations in costs and benefits. Despite the sensitivity, the economic benefit of haemodialysis improved with decreasing costs, and the BCR remained above one in most scenarios, unlike the CCC’s BCR. The findings of the sensitivity analysis highlight a need for strategies to reduce the cost of haemodialysis, such as controlling inflation, improving local production of haemodialysis commodities, and increasing the efficiency of the government’s medical products supply system. Sensitivity analysis enhances the credibility of economic evaluation and contributes to more informed healthcare decision-making in resource-limited settings [26]. Although the benefit-cost ratio was high when the study considered only the patient cost, this finding must be interpreted cautiously, as the current study included only the patients’ direct costs. Other scholars recommend accounting for the loss of productivity as an opportunity cost in economic analyses such as cost-benefit analyses [27]. All things being equal, including patients’ indirect costs will increase the cost and decrease the BCR.

The study contributes to the existing scholarship on cost-benefit analysis of different modalities for managing chronic kidney disease in LMICs. The current study is Nigeria’s first cost-benefit analysis of haemodialysis among ESKD patients since there is no other study on cost-benefit analysis of haemodialysis and CCC in Nigeria. Overall, our study’s findings on the cost-benefit analysis of haemodialysis can guide policy decisions on integrating haemodialysis fully into social health insurance benefits, enabling all socioeconomic groups to have affordable access to life-saving haemodialysis treatment. Nevertheless, our study has some limitations. The cost and willingness to pay studies relied on patients’ self-reports, which are prone to under- or over-reporting. Also, the costing studies excluded patients’ indirect costs, such as productivity losses [28]. Nevertheless, our study followed recommended costing and WTP practices to determine the costs and maximum WTP. WTP is the ideal approach for estimating non-marketed benefits in economic evaluations despite some limitations [21].

## Conclusion

This study estimated the social value of funding haemodialysis for managing ESKD patients in Abuja, Nigeria, using the cost of illness and willingness to pay approaches. The positive net benefits and benefit-cost ratios of haemodialysis demonstrate that the benefits the society derives from haemodialysis exceed that of comprehensive conservative care. Based on the social value of dialysis, the NHIA might expand coverage for haemodialysis in its benefits package. Decision-makers must also consider other policy options, such as increasing public subsidies to enrol people with low income into social health insurance schemes, negotiating capped payment rates for haemodialysis, and providing haemodialysis through public facilities at affordable costs.

### Electronic supplementary material

Below is the link to the electronic supplementary material.

Supplementary Material 1

## Data Availability

No datasets were generated or analysed during the current study.

## Abbreviations

BCR: Benefit–cost ratio
CBA: Cost–benefit analysis
CCC: Comprehensive conservative care
CKD: Chronic kidney disease
CVM: Contingent valuation method
ESKD: End–stage kidney disease
FCT: Federal Capital Territory
KRT: Kidney replacement therapy
LMICs: Low and middle–income countries
NHIA: National health insurance authority
OOP: Out–of–pocket payment
PPP: Public–private partnership
and WTP: Willingness to pay
