# Supplementary material for: Cost-benefit analysis of haemodialysis in patients with end-stage kidney disease in Abuja, Nigeria
Source: Health Econ Rev. 2024 Jul 3;14:47. doi: 10.1186/s13561-024-00529-z (PMC11221004; doi:10.1186/s13561-024-00529-z)
Supplement: Supplementary file 1 — Supplementary Material 1 [file 13561_2024_529_MOESM1_ESM.docx]

| **Table S1 Costing template for the study** | | | |
| --- | --- | --- | --- |
| Study arm |  | Cost elements | Unit cost (USD) |
| Haemodialysis (n =230) | 1 | Fixed costs (Machine, building and equipment) | 20.5 |
|  | 2 | Variable costs (personnel, drugs, supplies and overhead) | 103.2 |
|  | 3 | Total provider cost | 123.69 |
|  | 4 | Direct patient costs (out-of-pocket) | 28.51 |
|  | 5 | Total cost (provider and patient) | 152.2 |
|  |  |  |  |
| Comprehensive conservative care (n = 210) | 1 | Fixed costs (Building and equipment) | 30.11 |
|  | 2 | Variable costs (Staff, drugs, supplies and overhead) | 8.33 |
|  | 3 | Total provider cost | 38.85 |
|  | 4 | Direct patient costs (out-of-pocket for transport, drugs, laboratory and feeding) | 33.59 |
|  | 5 | Total cost (provider and patient) | 72.44 |
